# A randomized phase II study of gemcitabine plus Z-360, a CCK2 receptor-selective antagonist, in patients with metastatic pancreatic cancer as compared with gemcitabine plus placebo

**DOI:** 10.1007/s00280-017-3351-4

**Published:** 2017-06-20

**Authors:** Makoto Ueno, Chung Pin Li, Masafumi Ikeda, Hiroshi Ishii, Nobumasa Mizuno, Taketo Yamaguchi, Tatsuya Ioka, Do Youn Oh, Wataru Ichikawa, Takuji Okusaka, Yutaka Matsuyama, Daichi Arai, Li Tzong Chen, Young Suk Park, Junji Furuse

**Affiliations:** 1 Division of Hepatobiliary and Pancreatic Medical Oncology, Kanagawa Cancer Center, 2-3-2, Nakao, Asahi-ku, Yokohama-shi, Kanagawa 241-8515 Japan; 2 Division of Gastroenterology and Hepatology, Department of Medicine, Taipei Veterans General Hospital, 201, Section 2, Shin-Pai Road, Taipei, 11217 Taiwan; 3 School of Medicine, National Yang-Ming University, 155, Section 2, Linong Street, Taipei, 112 Taiwan; 4 Department of Hepatobiliary and Pancreatic Oncology, National Cancer Center Hospital East, 6-5-1, Kashiwanoha, Kashiwa-shi, Chiba 277-8577 Japan; 5 Department of Gastroenterology, Cancer Institute Hospital, Japanese Foundation for Cancer Research, 3-8-31, Ariake, Koto-ku, Tokyo 135-8550 Japan; 6 Department of Gastroenterology, Aichi Cancer Center Hospital, 1-1 Kanokoden, Chikusa-ku, Nagoya, Aichi 464-8681 Japan; 7 Department of Gastroenterology, Chiba Cancer Center, 666-2 Nitona-cho, Chuo-ku, Chiba-shi, Chiba 260-8717 Japan; 8 Department of Gastrointestinal Cancer Screening and Surveillance, Osaka Medical Center for Cancer and Cardiovascular Disease, 3-3 Nakamichi 1-Chome, Higashinari-ku, Osaka 537-8511 Japan; 9 Division of Medical Oncology, Department of Internal Medicine, Seoul National University Hospital, Cancer Research Institute, Seoul National University College of Medicine, 101 Daehak-ro, Jongno-gu, Seoul, 03080 South Korea; 10 Division of Medical Oncology, Department of Medicine, Showa University School of Medicine, 1-30 Fujigaoka, Aoba-ku, Yokohama, Kanagawa 227-8501 Japan; 11 Department of Hepatobiliary and Pancreatic Oncology, National Cancer Center Hospital, 5-1-1, Tsukiji, Chuo-ku, Tokyo 104-0045 Japan; 12 Department of Biostatistics, School of Public Health, The University of Tokyo, 7-3-1 Hongo, Bunkyo-ku, Tokyo Japan; 13 Division of Clinical Research 3, ZERIA Pharmaceutical Co., Ltd., 10-11, Nihonbashi Kobuna-cho, Chuo-ku, Tokyo 103-8351 Japan; 14 National Institute of Cancer Research, National Health Research Institutes, 367, Sheng-Li Rd., North District, 70456 Tainan, Taiwan; 15 Division of Hematology-Oncology, Department of Medicine, Samsung Medical Center, Sungkyunkwan University School of Medicine, 50, Irwon-Dong, Gangnam-Gu, Seoul, 06351 South Korea; 16 Department of Internal Medicine, Medical Oncology, Kyorin University School of Medicine, 6-20-2, Shinkawa, Mitaka-shi, Tokyo 181-8611 Japan; 17 Department of Gastroenterology, Clinical Research Center, National Hospital Organization Shikoku Cancer Center, 160, Kou, Minamiumemoto-machi, Matsuyama-shi, Ehime 791-0280 Japan; 18 Department of Gastrointestinal Cancer Screening and Surveillance, Osaka International Cancer Institute, 3-1-69, Otemae, Chuo-ku, Osaka, 541-8567 Japan

**Keywords:** Clinical trial, Phase II, Pancreatic cancer, Metastatic, Gemcitabine, Cholecystokinin

## Abstract

**Background:**

We investigated the efficacy and safety of 60, 120, or 240 mg of Z-360, which is a highly potent cholecystokinin2-receptor-selective antagonist, combined with gemcitabine in patients with metastatic pancreatic cancer.

**Methods:**

Patients were randomly assigned in a 1:1:1:1 ratio to one of four treatment groups. Patients received 1000 mg/m^2^ gemcitabine for each cycle and Z-360 tablets of 60 mg (GZ 60 mg group), 120, 240 mg or placebo tablets (Gem group) orally twice daily. The primary endpoint was overall survival (OS).

**Results:**

The median OS was 1.3 months longer in the GZ 60 mg group compared with the Gem group (8.5 vs. 7.2 months) and the risk of death was reduced by 19% compared with the Gem group, although there were no statistically significant differences. The study treatments were well tolerated.

**Conclusions:**

In this Phase II study, no statistically significant differences between the GZ groups and Gem group were detected in any analysis. However, Z-360 in dose of 60 mg tends to improve OS in patients with metastatic pancreatic cancer with low toxic effect. Further exploratory trials with other agents such as gemcitabine plus nab-paclitaxel might be beneficial.

**Electronic supplementary material:**

The online version of this article (doi:10.1007/s00280-017-3351-4) contains supplementary material, which is available to authorized users.

## Introduction

Pancreatic cancer (PC) is the eighth leading cause of cancer-related mortality worldwide [1], and the majority of patients do not have curable disease. Since 1997, gemcitabine therapy has been the standard first-line chemotherapy for patients with unresectable locally advanced or metastatic PC; however, 1-year survival rates of patients with metastatic disease are only approximately 20% [2, 3]. FOLFIRINOX has become one of the standard regimens for metastatic PC. However, the use of FOLFIRINOX is limited to patients in good medical condition because of high toxicity [2]. Gemcitabine plus nab-paclitaxel is another treatment option for patients in fair medical condition [3]. However, outcomes remain poor, and some patients cannot receive such intensive chemotherapy. There is thus an urgent need to develop other less toxic treatment options that have other mechanisms of action.

The gastrointestinal peptides gastrin and cholecystokinin (CCK) have been found to stimulate the growth of several human pancreatic cancer cell lines in culture and pancreatic xenograft rodent models [4]. Because the proliferation of PC cells has been reported to be mediated by CCK2 receptor, this receptor has been suggested to be a potential therapeutic target for PC [5]. CCK-2 receptor downregulation can also enhance the apoptosis of pancreatic cancer cells [6].

Z-360 is a novel, orally active, and highly potent CCK2-receptor-selective antagonist. Preclinical studies have shown that Z-360 either alone or in combination with gemcitabine can inhibit the growth of PC in animal xenograft models [7].

In a randomized phase Ib/IIa study, oral Z-360 at dose of 120 or 240 mg twice daily combined with standard, weekly gemcitabine 1000 mg/m^2^ was well tolerated in patients with advanced PC. More patients receiving Z-360 reported improvement in pain than those receiving gemcitabine alone. In addition, patients who received Z-360 120 mg twice daily had better survival than those who received Z-360 240 mg twice daily or gemcitabine alone [8]. To confirm and extend the results of that study, we investigated the efficacy and safety of 60, 120, or 240 mg of Z-360 twice daily combined with gemcitabine in this phase II study.

## Patients and methods

### Study design

This phase II multicenter, randomized, double-blind, parallel group, placebo-controlled study, sponsored by ZERIA Pharmaceutical Co., Ltd., Tokyo, Japan, was conducted at 27 sites in Japan, South Korea, and Taiwan in accordance with the Declaration of Helsinki and the International Conference on Harmonization Consolidated Guideline E6 for Good Clinical Practice. Written informed consent for participation in the study was obtained from all patients. Patients were randomly assigned in a 1:1:1:1 ratio to receive gemcitabine plus placebo (Gem group), Z-360 60 mg (GZ 60 mg group), Z-360 120 mg (GZ 120 mg group), or Z-360 240 mg (GZ 240 mg group). Randomization was performed according to a computer-generated schedule and carried out by independent interactive web response system. Patients were dynamically assigned to the treatment groups in a stochastic manner. The allocations factors were the Eastern Cooperative Oncology Group (ECOG) performance status (PS) (0 + 1 vs. 2), history of systemic chemotherapy for metastatic PC (yes vs. no), and site per country.

### Patients

Eligible adults (20 years of age or older) had histological or cytological evidence of metastatic PC (either measurable or non-measurable disease according to the Response Evaluation Criteria in Solid Tumors [RECIST], version 1.1.), an ECOG PS 0–2, a life expectancy of ≥12 weeks, and adequate hematologic, hepatic, and renal functions. Both chemo-naïve patients and patients who received any previous systemic chemotherapy (except gemcitabine) prior to 4 weeks before randomization were eligible (Supplementary Text 1). Patient’s eligibility was determined by each investigator.

### Treatment

Patients received gemcitabine in a dose of 1000 mg/m^2^ as a 30-min intravenous infusion once weekly for 3 weeks of each 28-day cycle Dose interruption or reduction of gemcitabine was performed according to predefined criteria (Supplementary Text 2). Patients received Z-360 tablets in a fixed dose of 60, 120, or 240 mg or matching placebo tablets orally twice daily until disease progression, unacceptable toxic effects, or the withdrawal of consent.

### Assessments

Patient’s conditions were assessed on each visit to receive gemcitabine. All adverse events (AEs) were evaluated according to the National Cancer Institute Common Terminology Criteria for Adverse Events, version 4.0. Tumor lesions were assessed by computed tomography or magnetic resonance imaging every 6 weeks until the treatment discontinuation criteria were met. The attending physicians radiologically assessed disease progression according to study-specific modified RECIST (Supplementary Text 3). Clinical disease progression was also assessed on the basis of the patient’s global status. An independent review committee (IRC) assessed radiological disease progression according to RECIST, version 1.1. Progression-free survival (PFS) was calculated on the basis of the date of radiological disease progression as evaluated by the IRC. Clinical disease progression as evaluated by each investigator was also used as an event when calculating PFS.

### Endpoints

The primary endpoint was overall survival (OS), defined as the time from the date of randomization to the date of death. If a patient was alive at the end of the study or was lost to follow-up, data on OS were censored. Secondary endpoints were PFS, response rate (RR), disease control rate (DCR), quality of life (QOL) score (Supplementary Text 4), and AEs.

### Statistical analysis

The required sample size was estimated on the basis of the following assumptions: a hazard ratio (HR) of ‘1:0.70:0.70:0.70’ (Gem group:GZ 60 mg group:GZ 120 mg group:GZ 240 mg group), a 12-month recruitment period and a 12-month follow-up period, and a 30% 1-year survival rate in the Gem group. At a significance level (one-sided) of 20%, a Cox proportional-hazards model was used to compare the difference in survivor function between the Gem group and pooled GZ group (pooled data from the GZ 60 mg, GZ 120 mg, and GZ 240 mg groups) by using a contrast ratio of −1:1/3:1/3:1/3. A power of 81.6% was achieved according to the results of simulation analysis with 40 patients in each treatment group. It was estimated that 118 events would be needed to achieve the power.

The full analysis set (FAS) included randomized patients who received at least one dose of the study drug and for whom any information on post-baseline survival status was available. The safety set (SS) included all patients who received at least one dose of the study drug. The FAS was used for all efficacy evaluations and the SS was used for all safety evaluations.

Time-to-event variables such as OS, PFS, and survival rate and corresponding 2-sided 95% CI for each treatment group were analyzed with the Kaplan–Meier method. The HR of time-to-event variables and the corresponding 2-sided 95% CI were determined with a Cox proportional hazards model including treatment group, ECOG PS (0 + 1 vs. 2), and previous treatment for metastatic PC (yes vs. no) as covariates, with a baseline hazard unique to each country. Subgroup analyses were performed without any adjustment.

The responses to the European Organization for Research and Treatment of Cancer (EORTC) Quality of Life Questionnaire Core 30 (QLQ-C30) questionnaires were analyzed in accordance with the EORTC guidelines [9]. Repeated-measures linear regression analysis was performed with the use of a mixed model to assess the QOL endpoint. The model used for the analysis of QOL included the explanatory variables of treatment group, baseline score, ECOG PS (0 + 1 vs. 2), previous history of systemic chemotherapy for metastatic PC (yes vs. no), visit and country, and visit by treatment interaction. The analysis plan was finalized before breaking the key code, and the statistical analyses were performed at Zeria Pharmaceutical Co., Ltd. All statistical calculations were performed with the use of SAS, Release 9.3 (SAS Institute Inc., Cary, NC, USA).

## Results

### Patients

**Fig. 1:**
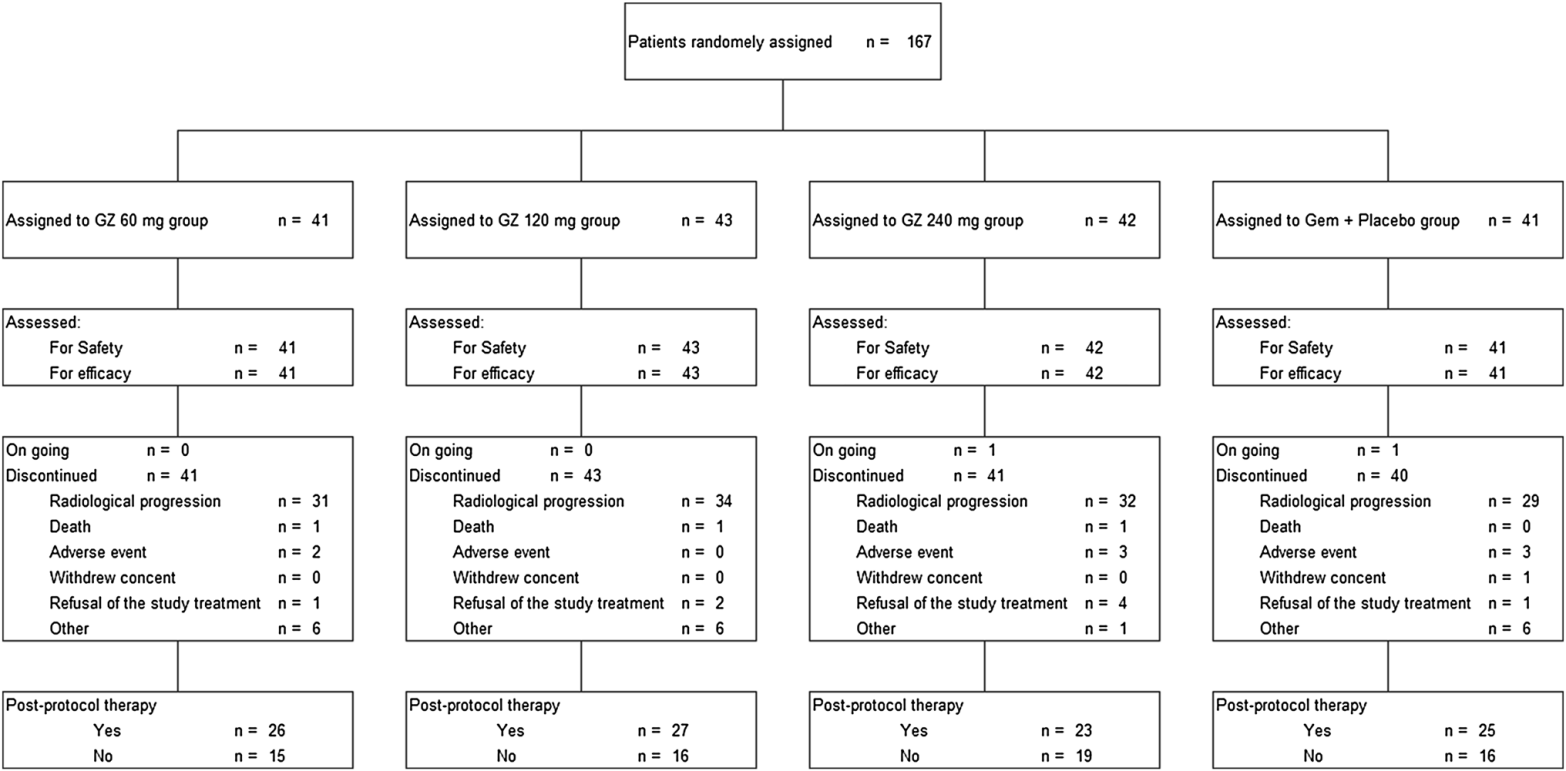
CONSORT diagram

A total of 167 patients (95 in Japan, 44 in South Korea, 28 in Taiwan) were randomly assigned to treatment between April 2014 and November 2014. The data cut-off date was November 30, 2015. All randomized patients (41, 43, 42, and 41 patients in the GZ 60 mg, GZ 120 mg, GZ 240 mg, and Gem groups, respectively) were included in the FAS and SS populations (Fig. 1). No patient violated the eligibility criteria. Few patients in each group had a PS of 2 or a history of systemic chemotherapy for metastatic PC. The proportion of patients with a neutrophil-to-lymphocyte ratio (NLR) of >4.0, which was reported to be a prognostic factor in advanced PC [10], was higher in the GZ 240 mg group than in the other groups (Table 1).

**Table 1:** Patient characteristics (full analysis set)

Characteristic	GZ 60 mg (*N* = 41) *n* (%)	GZ 120 mg (*N* = 43) *n* (%)	GZ 240 mg (*N* = 42) *n* (%)	Gem + placebo (*N* = 41) *n* (%)
Age (years)				
Mean	63.7	66.7	65.2	64.6
Median	66.0	68.0	67.0	65.0
<65 (years)	19 (46.3)	16 (37.2)	18 (42.9)	19 (46.3)
≥65 (years)	22 (53.7)	27 (62.8)	24 (57.1)	22 (53.7)
Gender				
Male	26 (63.4)	24 (55.8)	24 (57.1)	22 (53.7)
Female	15 (36.6)	19 (44.2)	18 (42.9)	19 (46.3)
Body surface area (m^2^)				
Mean	1.589	1.553	1.531	1.569
Median	1.548	1.553	1.575	1.579
Eastern Cooperative Oncology Group Performance Status^a^				
0	19 (46.3)	18 (41.9)	21 (50.0)	22 (53.7)
1	22 (53.7)	22 (51.2)	19 (45.2)	18 (43.9)
2	0 (0.0)	3 (7.0)	2 (4.8)	1 (2.4)
CA19-9 level^b^				
Mean	5682.7	6693.6	6704.5	5013.2
Median	669.0	682.6	614.4	349.6
Neutrophil lymphocyte ratio				
≤4	32 (78.0)	35 (81.4)	24 (57.1)	29 (72.5)
>4	9 (22.0)	8 (18.6)	18 (42.9)	11 (27.5)
Previous treatment for metastatic PA				
Yes	5 (12.2)	7 (16.3)	5 (11.9)	5 (12.2)
No	36 (87.8)	36 (83.7)	37 (88.1)	36 (87.8)
Diagnostic information				
Initial occurrence	36 (87.8)	35 (81.4)	32 (76.2)	33 (80.5)
Reoccurrence	5 (12.2)	8 (18.6)	10 (23.8)	8 (19.5)

^a^Eastern Cooperative Oncology Group Performance Status range from 0 to 4, with lower scores indicating better performance status

^b^A total of 166 patients had a baseline CA19-9 measurement. 1 patient in Gem + placebo group was excluded from the analysis due to missing data

### Efficacy

**Fig. 2:**
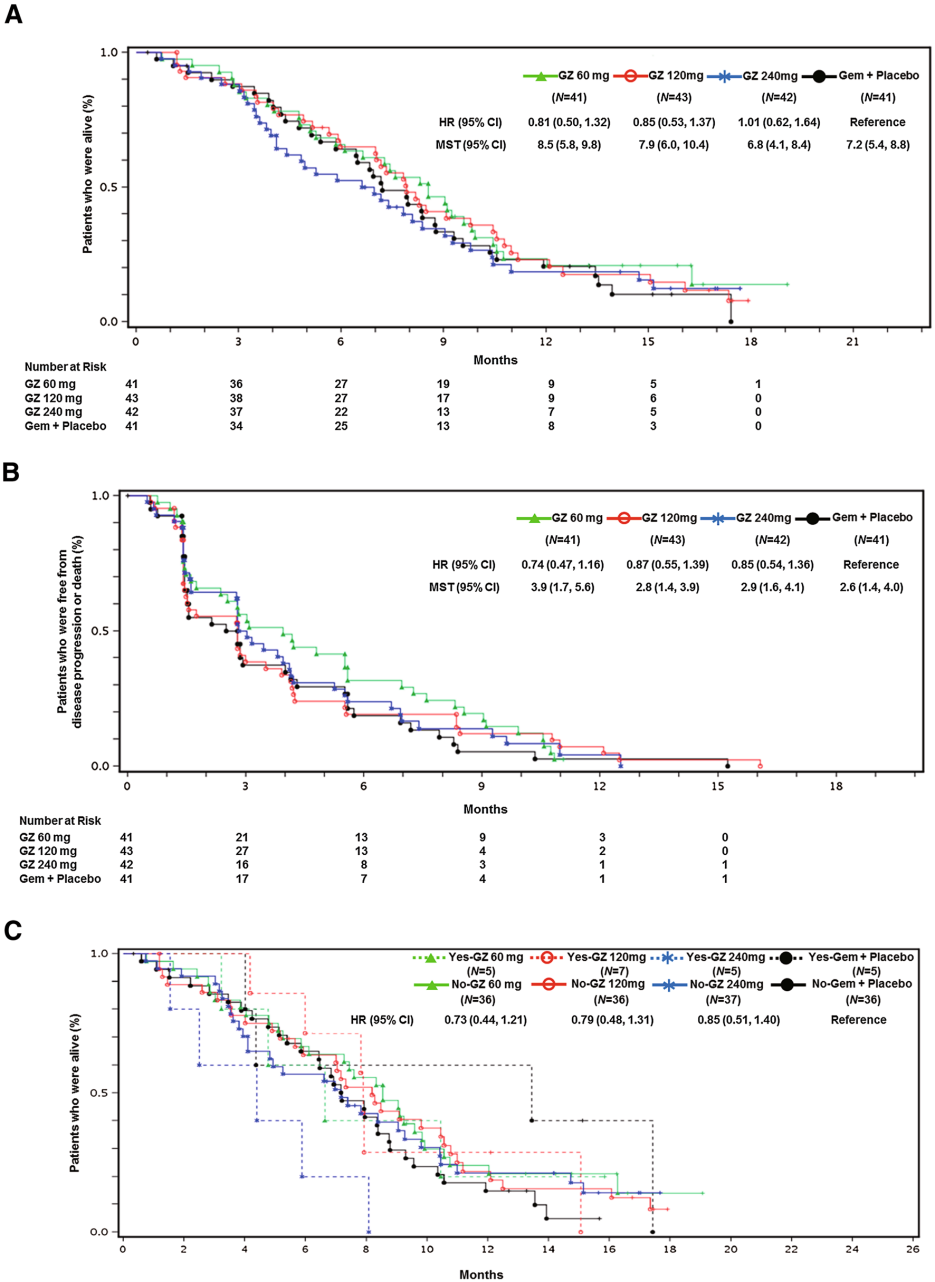
Kaplan–Meier estimates of **a** overall survival, **b** progression-free survival (assessed by an independent review committee) and **c** overall survival in a subgroup analysis of previous treatment for metastatic PC (yes vs. no) (full analysis set)

The number of events was 140 (84% of patients). The median survival (95% CI) was 8.5 months (5.8–9.8), 7.9 months (6.0–10.4), 6.8 months (4.1–8.4), and 7.2 months (5.4–8.8) in the GZ 60 mg, GZ 120 mg, GZ 240 mg, and Gem groups, respectively (Fig. 2a). The HR for OS (95% CI) was 0.89 (0.60–1.32) in the pooled Z-360 group and 0.81 (0.50–1.32), 0.85 (0.53–1.37), and 1.01 (0.62–1.64) in the GZ 60 mg, GZ 120 mg, and GZ 240 mg groups, respectively. The efficacy of Z-360 in the subgroup of chemo-naïve metastatic PC was better than that of the overall patient population (HR in OS: 0.73, 0.79, and 0.85 in the GZ 60 mg, GZ 120 mg, and GZ 240 mg groups, respectively) (Fig. 2c; Supplementary Table 1). The 1-year survival rate (95% CI) was 23.4% (11.8–37.3), 23.1% (11.6–36.8), 18.6% (8.3–32.1), and 20.5% (9.7–34.3), in the GZ 60 mg, GZ 120 mg, GZ 240 mg, and Gem groups, respectively.

In the analysis of PFS, 161 patients (96.4%) had disease progression or died. The median PFS (95% CI) was 3.9 months (1.7–5.6), 2.8 months (1.4–3.9), 2.9 months (1.6–4.1), and 2.6 months (1.4–4.0) in the GZ 60 mg, GZ 120 mg, GZ 240 mg, and Gem groups, respectively (Fig. 2b). The HR for PFS (95% CI) was 0.82 (0.56–1.20) in the pooled Z-360 group, and 0.74 (0.47–1.16), 0.87 (0.55–1.39), and 0.85 (0.54–1.36) in the GZ 60 mg, GZ 120 mg, and GZ 240 mg groups, respectively.

Post-protocol anticancer therapy was given to 26 (63%), 27 (63%), 23 (55%), and 25 (61%) patients in the GZ 60 mg, GZ 120 mg, GZ 240 mg, and Gem groups, respectively. The most common therapy was S-1 (27% in total). Only a few patients received FOLFIRINOX (3% in total) or gemcitabine plus nab-paclitaxel (8% in total) as post-protocol anticancer therapy.

The response rates (95% CI) according to independent review were 9.8% (2.7–23.1%), 11.6% (3.9–25.1%), 9.5% (2.7–22.6%), and 2.4% (0.1–12.9%), in the GZ 60 mg, GZ 120 mg, GZ 240 mg, and Gem groups, respectively. There were no major differences in DCR among the groups. In pre-specified subgroup analysis, the efficacy of Z-360 in the patients with chemo-naïve metastatic PC was better than that in the group as a whole (Fig. 2c; Supplementary Table 1).

Baseline carbohydrate antigen 19-9 (CA19-9) levels were measured in 166 patients. A total of 59, 58, 38, and 34% of the patients had a reduction of ≥20% from the baseline level in the GZ 60 mg, GZ 120 mg, GZ 240 mg, and Gem groups, respectively.

The responses to the QLQ-C30 questionnaire at baseline were evaluated for all patients in the FAS population. The scores for “Global Health Status and Quality of Life scale” and “scale for pain” deteriorated at week 4 in all groups, but at week 12 both scales recovered to the same level as that at baseline in GZ 60 mg and GZ 120 mg groups. In contrast, both scales deteriorated in a similar manner in the GZ 240 mg and Gem groups (Supplementary Table 2). No major differences were noted among the groups in the other variables.

### Safety

Only 4 treatment-related serious adverse events (SAEs) were reported: cellulitis in the GZ 60 mg group, upper gastrointestinal hemorrhage and *Pneumocystis jirovecii* pneumonia in the GZ 240 mg group, and vomiting in the Gem group. Fatal events, excluding progression of PC, were pulmonary embolism in the GZ 60 mg group, acute respiratory failure in the GZ 120 mg group, and pneumonia aspiration, cardiac failure, and small intestinal obstruction in the GZ 240 mg group. The proportion of patients with SAEs or with AEs leading to discontinuation of the study treatment did not differ among the groups. AEs that occurred in more than 10% of patients in any treatment group are shown in Table 2. Hematological events known as adverse reactions of gemcitabine, such as decreased neutrophil count were reported more often in the GZ 60 mg and GZ 120 mg groups than in the other groups. However, no febrile neutropenia was reported in any group. In contrast, fatigue, asthenia, and decreased appetite were reported more often in the GZ 240 mg group than in the other groups.

**Table 2:** Treatment-emergent adverse events reported by ≥10% of patients in any treatment group and receipt of immunostimulants (safety set)

	GZ 60 mg (*N* = 41) *n* (%)	GZ 120 mg (*N* = 43) *n* (%)	GZ 240 mg (*N* = 42) *n* (%)	Gem + placebo (*N* = 41) *n* (%)
Hematological analysis				
Platelet count decreased	17 (41.5)	20 (46.5)	10 (23.8)	11 (26.8)
Neutrophil count decreased	21 (51.2)	13 (30.2)	14 (33.3)	9 (22.0)
≥Grade 3	16 (39.0)	12 (27.9)	13 (31.0)	9 (22.0)
Neutropenia	2 (4.9)	5 (11.6)	2 (4.8)	3 (7.3)
≥Grade 3	2 (4.9)	4 (9.3)	2 (4.8)	2 (4.9)
White blood cell count decreased	13 (31.7)	14 (32.6)	10 (23.8)	7 (17.1)
Anemia	16 (39.0)	15 (34.9)	7 (16.7)	12 (29.3)
Receipt of immunostimulants	3 (7.3)	2 (4.7)	3 (7.1)	4 (9.8)
Non-hematological analysis				
Weight decreased	6 (14.6)	4 (9.3)	1 (2.4)	0 (0.0)
Nausea	16 (39.0)	19 (44.2)	15 (35.7)	15 (36.6)
Constipation	17 (41.5)	11 (25.6)	8 (19.0)	10 (24.4)
Vomiting	10 (24.4)	10 (23.3)	8 (19.0)	11 (26.8)
Diarrhea	4 (9.8)	16 (37.2)	6 (14.3)	6 (14.6)
Ascites	4 (9.8)	8 (18.6)	5 (11.9)	3 (7.3)
Stomatitis	5 (12.2)	3 (7.0)	1 (2.4)	4 (9.8)
Pyrexia	12 (29.3)	10 (23.3)	9 (21.4)	12 (29.3)
Fatigue	8 (19.5)	5 (11.6)	11 (26.2)	8 (19.5)
Edema peripheral	6 (14.6)	8 (18.6)	5 (11.9)	2 (4.9)
Malaise	3 (7.3)	6 (14.0)	2 (4.8)	8 (19.5)
Asthenia	3 (7.3)	2 (4.7)	6 (14.3)	3 (7.3)
Decreased appetite	16 (39.0)	19 (44.2)	22 (52.4)	9 (22.0)
Hypoalbuminemia	3 (7.3)	5 (11.6)	4 (9.5)	1 (2.4)
Rash	2 (4.9)	5 (11.6)	7 (16.7)	6 (14.6)
Rash maculo-papular	3 (7.3)	2 (4.7)	2 (4.8)	5 (12.2)
Nasopharyngitis	2 (4.9)	1 (2.3)	5 (11.9)	0 (0.0)
Cancer pain	3 (7.3)	3 (7.0)	5 (11.9)	3 (7.3)
Insomnia	5 (12.2)	4 (9.3)	4 (9.5)	3 (7.3)

High exposure to the study drug and high relative dose intensity (RDI) (>90%) of gemcitabine were maintained in all treatment groups.

## Discussion

In this multicenter, placebo-controlled, randomized, phase II study, no statistically significant differences between the GZ groups and Gem group were detected in any analysis. However, Z-360 tends to improve OS and PFS in patients with metastatic PC.

The median OS and PFS were prolonged by 1.3 months in the GZ 60 mg group as compared with the respective values in the Gem group. In the GZ 60 mg group, a 19% reduction in the risk of death, and a 26% reduction in the risk of disease progression or death as compared with the Gem group were observed. Higher proportions of patients in the GZ 60 mg and GZ 120 mg groups had reductions of ≥20% in serum CA19-9 levels, which has been reported to be associated with superior survival [11], as compared with the gemcitabine group, and reduced CA19-9 secretion might indicate cytotoxicity associated with cell apoptosis. This result was similar to that in a pivotal study of gemcitabine plus nab-paclitaxel (proportions of patients who had reductions of ≥20% in serum CA19-9 levels: 61% [gemcitabine plus nab-paclitaxel group] versus 44% [gemcitabine group]) [3].

The study treatments were well tolerated, and high exposure to the study drug and high RDI (>90%) of gemcitabine were maintained in all treatment groups. Therefore, this treatment is considered quite manageable.

Gemcitabine plus Z-360 was associated with few safety concerns as compared with FOLFIRINOX or gemcitabine plus nab-paclitaxel. For example, the proportions of patients with a decrease in neutrophil count/neutropenia ≥Grade 3 in each GZ group in this study (Table 2) were low as compared with the results of a pivotal study in Japan of gemcitabine plus nab-paclitaxel (neutropenia: 70.6%) [12]. In addition, no febrile neutropenia was reported in any GZ group (gemcitabine plus nab-paclitaxel: 5.9%).

Moreover, the scores on the “Global Health Status and Quality of Life scale” and “scales for pain” at 12 weeks, reported to be important scales for the evaluation of PC [13], were maintained at the baseline levels in the GZ 60 mg and GZ 120 mg groups.

Z-360 thus can be easily given to patients who cannot tolerate highly cytotoxic treatment or who prefer to maintain their QOL.

The efficacy of Z-360 in subgroup of chemo-naïve metastatic PC was better than that of the overall patient population, which should be considered when conducting other trials in the future.

The OS in the GZ 60 mg and GZ 120 mg groups was better than that in the GZ 240 mg group, although the results for PFS were similar among the Z-360 groups. Higher proportion of patients has shown an NLR of >4.0 in GZ 240 mg group. AEs related to the patient’s global status, such as fatigue, asthenia, and decreased appetite, were reported more often in the GZ 240 mg group than in the other groups, which might have led to difficulty in administering subsequent therapies. Such factors might have resulted in the difference between OS and PFS.

Although our study had limitations because of its small sample size and exploratory nature, our results suggest that the efficacy of Z-360 might have reached a plateau at 60 mg twice daily. Because Z-360 is attributed to a different mechanism of action from that of currently available cytotoxic agents, it might also be beneficial to combine Z-360 with other agents, such as gemcitabine plus nab-paclitaxel. Recently, a CCK2 receptor-selective antagonist combined with an immune checkpoint inhibitor was reported to be effective in mice [14].

In conclusion, Z-360 tends to improve OS and PFS with low toxic effect in patients with metastatic PC. Further exploratory trials of 60 mg Z-360 twice daily with other agents might be beneficial.

## Electronic supplementary material

Below is the link to the electronic supplementary material.

Supplementary material 1 (PDF 231 kb)

## References

[1] Jemal A, Bray F, Center MM, Ferlay J, Ward E, Forman D (2011). Global cancer statistics. CA Cancer J Clin.

[2] Conroy T, Desseigne F, Ychou M, Bouché O, Guimbaud R, Bécouarn Y, Adenis A, Raoul J-L, Gourgou-Bourgade S, Cdl Fouchardière, Bennouna J, Bachet J-B, Khemissa-Akouz F, Péré-Vergé D, Delbaldo C, Assenat E, Chauffert B, Michel P, Montoto-Grillot C, Chem M, Ducreux M (2011). FOLFIRINOX versus gemcitabine for metastatic pancreatic cancer. N Engl J Med.

[3] Von-Hoff DD, Ervin T, Arena FP, Chiorean EG, Infante J, Moore M, Seay T, Tjulandin SA, Ma WW, Saleh MN, Harris M, Reni M, Dowden S, Laheru D, Bahary N, Ramanathan RK, Tabernero J, Hidalgo M, Goldstein D, Van-Cutsem E, Wei X, Iglesias J, Renschler MF (2013). Increased survival in pancreatic cancer with nab-paclitaxel plus gemcitabine. N Engl J Med.

[4] Clerc P, Leung-Theung-Long S, Wang TC, Dockray GJ, Bouisson M, Delisle M-B, Vaysse N, Pradayrol L, Fourmy D, Dufresne M (2002). Expression of CCK2 receptors in the murine pancreas: proliferation, transdifferentiation of acinar cells, and neoplasia. Gastroenterology.

[5] Rengifo-Cam W, Singh P (2004). Role of progastrins and gastrins and their receptors in GI and pancreatic cancers: targets for treatment. Curr Pharm Des.

[6] Fino KK, Matters GL, McGovern CO, Gilius EL, Smith JP (2012). Downregulation of the CCK-B receptor in pancreatic cancer cells blocks proliferation and promotes apoptosis. Am J Physiol Gastrointest Liver Physiol.

[7] Kawasaki D, Emori Y, Eta R, Iino Y, Hamano H, Yoshinaga K, Tanaka T, Takei M, Watson SA (2008). Effect of Z-360, a novel orally active CCK-2/gastrin receptor antagonist on tumor growth in human pancreatic adenocarcinoma cell lines in vivo and mode of action determinations in vitro. Cancer Chemother Pharmacol.

[8] Meyer T, Caplin ME, Palmer DH, Valle JW, Larvin M, Waters JS, Coxon F, Borbath I, Peeters M, Nagano E, Kato H (2009). A phase Ib/IIa trial to evaluate the CCK2 receptor antagonist Z-360 in combination with gemcitabine in patients with advanced pancreatic cancer. Eur J Cancer.

[9] Fayers P, Aaronson N, Bjordal K, Groenvold M, Curran D, Bottomley A (2001). EORTC QLQ-C30 scoring manual.

[10] Vivaldi C, Caparello C, Musettini G, Pasquini G, Catanese S, Fornaro L, Lencioni M, Falcone A, Vasile E (2016). First-line treatment with FOLFOXIRI for advanced pancreatic cancer in clinical practice: patients’ outcome and analysis of prognostic factors. J Cancer Clin Trials.

[11] Maisey NR, Norman AR, Hill A, Massey A, Oates J, Cunningham D (2005). CA19-9 as a prognostic factor in inoperable pancreatic cancer: the implication for clinical trials. Br J Cancer.

[12] Ueno H, Ikeda M, Ueno M, Mizuno N, Ioka T, Omuro Y, Nakajima TE, Furuse J (2016). Phase I/II study of nab-paclitaxel plus gemcitabine for chemotherapy-naive Japanese patients with metastatic pancreatic cancer. Cancer Chemother Pharmacol.

[13] Fayers P, Weeden S, Curran D (1998). EORTC QLQ-C30 reference values.

[14] Smith JP, Wang S, Reham A, Jablonski SA, Weiner LM (2016) Combination therapy with immune checkpoint inhibitor and CCK-receptor blockade increases survival of pancreatic cancer [abstract]. In: Proceedings of the 107th Annual Meeting of the American Association for Cancer Research (AACR), New Orleans, Louisiana, vol 76 (14 Suppl). AACR, p Abstract nr 4023

