# Supplementary material for: A randomized phase II study of gemcitabine plus Z-360, a CCK2 receptor-selective antagonist, in patients with metastatic pancreatic cancer as compared with gemcitabine plus placebo
Source: Cancer Chemother Pharmacol. 2017 Jun 20;80(2):307–15. doi: 10.1007/s00280-017-3351-4 (PMC5532401; doi:10.1007/s00280-017-3351-4)
Supplement: Supplementary file 1 — Supplementary material 1 (PDF 231 kb) [file 280_2017_3351_MOESM1_ESM.pdf]

Electronic supplementary material

Article title:

A randomized phase II study of gemcitabine plus Z-360, a CCK2 receptor selective antagonist, in patients with metastatic pancreatic cancer as compared with gemcitabine plus placebo

Journal name:

Cancer Chemotherapy and Pharmacology

Author names:

Makoto Ueno, Chung Pin Li, Masafumi Ikeda, Hiroshi Ishii, Nobumasa Mizuno, Taketo Yamaguchi, Tatsuya Ioka, Do Youn Oh, Wataru Ichikawa, Takuji Okusaka, Yutaka Matsuyama, Daichi Arai, Li Tzong Chen, Young Suk Park, Junji Furuse

Affiliation and e-mail address of the corresponding author:

Makoto Ueno, Division of Hepatobiliary and Pancreatic Medical Oncology, Kanagawa Cancer Center; E-mail: uenom@kcch.jp

Supplementary Appendix (online only)

All the investigators

In addition to the authors, the following principal investigators participated in our study: LY Bai, China Medical University & Hospital, Taichung, Taiwan; JS Chen, Chang Gung Medical Foudantion–Linkou Branch, Tao-Yuan, Taiwan; HJ Choi, Severance Hospital, Seoul, Korea; IJ Chung, Chonnam National University Hwasun Hsopital, South Korea, Jeonnam; C Hsu, National Taiwan University Hsopital, Taipei, Taiwan; HC Jeung, Gangnam Severance Hospital, Seoul, South Korea; JG Kim, Kyungpook National University Medical Center, Daegu, South Korea; JS Kim, Korea University Guro Hospital, Seoul, South Korea; SG Kim, Pusan National University Yangsan Hospital, Gyeongsangnam-do, South Korea; MH Lee, Inha University Hospital, Inchen, South Korea; J Lin, Mackay Memorial Hospital, Taipei, South Korea; H Maguchi, Teine-Keijinkai Hospital, Sapporo, Japan; Kun-Ming Rau, Chang Gung Medical Foudantion–Kaohsiung Branch, Kaohsiung, Taiwan; YS Shan, National Cheng Kung University Hospital, Tainan, Taiwan; HS Song, Keimyung University Dongsan Hospital, Dauge, South Korea; K Sugimori, Yokohama City Universiy Medical Center, Yokohama, Japan; H Ueno, National Cancer Center Hospital, Tokyo, Japan.

Supplementary Text 1 (online only)

Details of main enrollment criteria and exclusion criteria

Enrollment criteria:

Patients with histological or cytological evidence of metastatic Pancreatic Adenocarcinoma (Measurable disease or non-measurable disease by Response Evaluation Criteria in Solid Tumors [RECIST] version 1.1 criteria); Patients with a life expectancy of at least 12 weeks; Patients with an Eastern cooperative oncology group performance status (ECOG PS) of 0, 1 or 2; Patients with the following adequate organ functions: White blood cell count ≥3,000/μL (or absolute neutrophil count ≥1,500/μL) and ≤ 12,000/μL, Platelet count ≥100.0 × 10^9/L, Hemoglobin ≥9.0 g/dL, Serum creatinine ≤1.5 × the upper limit normal (ULN), Total bilirubin ≤2.0 × ULN, Serum aspartate transaminase levels ≤3.0 × ULN (≤5.0 × ULN in presence of liver metastases), and Serum alanine aminotransferase levels ≤3.0 × ULN (≤5.0 × ULN in presence of liver metastases); Patients who are able to take Z-360 orally, Patients with an age ≥20 years on the day the informed consent is signed.

Exclusion Criteria:

Patients received the following previous therapies for PA: Surgery within the 4 weeks prior to randomization, Radiation and chemoradiation within the 12 weeks prior to randomization, Radiation for pain relief within the 4 weeks prior to randomization, Gemcitabine used as a neoadjuvant or an adjuvant on surgery within the 24 weeks prior to randomization, Chemotherapy except Gemcitabine

used as an adjuvant on surgery within the 4 weeks prior to randomization, Gemcitabine ≥600 mg/m^2 as sensitizer for chemoradiation, Gemcitabine <600 mg/m^2 as sensitizer for chemoradiation within the 12 weeks prior to randomization, Gemcitabine used for systemic chemotherapy, or Systemic chemotherapies except Gemcitabine within the 4 weeks prior to randomization.

Supplementary Text 2 (online only)

Criteria of Dose interruption or reduction of gemcitabine or study drug

**Interruption of gemcitabine**	
Items	criteria
White blood cell count	<2,000/μL
Platelet count	<70 × 10^9/L
Investigator’s opinion	In the investigator’s opinion, Gemcitabine administration should be put on hold.
**Reduction of gemcitabine**	
White blood cell decreased	<1,000/μL (Grade 4: CTCAE ver. 4.0)
Neutrophil count decreased	<500/μL (Grade 4: CTCAE ver. 4.0)
Febrile neutropenia	Grade 3-4: CTCAE ver. 4.0
Platelet count decreased	<25 × 10^9/L (Grade 4: CTCAE ver. 4.0)
Rash maculo-papular	Grade 3: CTCAE ver. 4.0
Stevens-Johnson syndrome	Grade 4: CTCAE ver. 4.0
Others	Development of study drug related AEs causing hold for two consecutive weeks
**Interruption or reduction of study drug**	
At the discretion of the investigator based was permitted as required in patients with AEs.	

CTCAE: Common Terminology Criteria for Adverse Events

Supplementary Text 3 (online only)

Definition of radiological progression in study treatment discontinuance critera

Radiological progression:

Investigators will judge "progression" based on the following criteria: Progressive Disease (PD) for measurable lesions defined as at least a 20% increase in the sum of diameters, taking as reference the baseline sum. In addition to the relative increase of 20%, the sum must also demonstrate an absolute increase of at least 5 mm; PD for non-measurable lesions defined as sufficient to require a change in therapy (e.g., an increase in tumor burden representing an additional 73% increase in 'volume'); Appearance of one or more new lesions.

Supplementary Text 4 (online only)

Secondary Outcome Measures

The secondary endpoints for this study are safety and tolerance, Progression Free Survivlal (PFS), Response Rate (RR), Disease Control Rate (DCR) and QoL (European Organization for Research and Treatment of Cancer [EORTC] Quality of Life Questionnaire Core 30 [QLQ-C30]). Progression free survival is to be based on MRI or CT results (central reviewed results) obtained every 6 weeks using RECIST ver. 1.1 criteria and analyzed statistically with the same method for OS. Data on patients who were lost to follow-up or who discontinued treatment before confirmation of disease progression were censored on the last day of tumor assessment.

The RR is composed of CR plus PR. The DCR is composed of CR plus PR plus SD.

Supplementary Table 1. Overall Survival Analysis (FAS) – Subgroup

	Group	No.of Patients	MST (month) [95% CI]	Cox Proportional Hazards Model Hazard ratio [95% CI]
**Previous Treatment For Metastatic PC**				
Yes	GZ 60 mg	5	6.6 [3.2, NA]	1.80 [0.40, 8.15]
	GZ 120 mg	7	7.9 [4.2, 15.0]	1.81 [0.44, 7.49]
	GZ 240 mg	5	4.4 [1.5, 8.1]	4.88 [1.06, 22.43]
	Gem + Placebo	5	13.4 [4.0, 17.4]	[reference]
No	GZ 60 mg	36	8.5 [5.8, 9.8]	0.73 [0.44, 1.21]
	GZ 120 mg	36	8.2 [5.7, 10.4]	0.79 [0.48, 1.31]
	GZ 240 mg	37	7.2 [4.1, 9.3]	0.85 [0.51, 1.40]
	Gem + Placebo	36	7.2 [5.4, 8.7]	[reference]
**Age (years)**				
< 65	GZ 60 mg	19	8.5 [5.1, 10.7]	0.73 [0.36, 1.50]
	GZ 120 mg	16	7.2 [4.2, 12.5]	0.90 [0.43, 1.87]
	GZ 240 mg	18	7.0 [4.1, 14.7]	0.82 [0.39, 1.72]
	Gem + Placebo	19	8.3 [4.4, 9.3]	[reference]
≥ 65	GZ 60 mg	22	8.4 [3.8, 9.9]	0.91 [0.48, 1.73]
	GZ 120 mg	27	8.3 [5.2, 10.5]	0.93 [0.51, 1.69]
	GZ 240 mg	24	5.8 [3.6, 9.0]	1.37 [0.73, 2.54]
	Gem + Placebo	22	6.8 [5.1, 10.5]	[reference]
**Gender**				
Male	GZ 60 mg	26	7.5 [4.8, 9.8]	1.02 [0.57, 1.84]
	GZ 120 mg	24	8.3 [4.9, 10.8]	0.84 [0.46, 1.54]
	GZ 240 mg	24	4.6 [3.5, 7.4]	1.15 [0.62, 2.11]
	Gem + Placebo	22	7.9 [4.0, 9.6]	[reference]
Female	GZ 60 mg	15	9.2 [3.2, NA]	0.55 [0.23, 1.27]
	GZ 120 mg	19	7.3 [5.2, 10.5]	0.96 [0.47, 1.98]
	GZ 240 mg	18	8.4 [4.9, 11.0]	0.88 [0.42, 1.85]
	Gem + Placebo	19	6.9 [5.1, 13.4]	[reference]
**ECOG PS**				
0+1	GZ 60 mg	41	8.5 [5.8, 9.8]	0.81 [0.51, 1.31]
	GZ 120 mg	40	7.9 [7.0, 10.5]	0.83 [0.52, 1.33]
	GZ 240 mg	40	6.9 [4.1, 9.0]	1.00 [0.62, 1.61]
	Gem + Placebo	40	7.2 [5.4, 8.8]	[reference]
2	GZ 60 mg	0	NA	NA
	GZ 120 mg	3	3.5 [1.2, 8.3]	NA
	GZ 240 mg	2	5.2 [3.5, 7.0]	NA
	Gem + Placebo	1	NA	[reference]
**Diagnostic Information of PC**				
Initial Occurrence	GZ 60 mg	36	8.4 [5.8, 9.8]	0.88 [0.53, 1.48]

	GZ 120 mg	35	7.3 [5.7, 8.3]	0.99 [0.60, 1.65]
	GZ 240 mg	32	6.4 [3.8, 8.4]	1.19 [0.70, 2.01]
	Gem + Placebo	33	7.9 [4.8, 9.6]	[reference]
Reccurrence	GZ 60 mg	5	9.1 [4.0, NA]	0.51 [0.13, 1.99]
	GZ 120 mg	8	12.1 [3.1, 12.5]	0.53 [0.17, 1.67]
	GZ 240 mg	10	7.3 [0.7, 15.1]	0.72 [0.25, 2.08]
	Gem + Placebo	8	6.8 [3.4, 9.3]	[reference]

MST: Median Survival Time, ECOG PS: Eastern Cooperative Oncology Group performance Status,
PC: Pancreatic cancer

Supplementary Table 2. Analysis of QLQ C-30 (FAS)

	GZ 60 mg (*N*=41)	GZ 120 mg (*N*=43)	GZ 240 mg (*N*=42)	Gem + Placebo (*N*=41)
**Global Health Status Change from Baseline** *				
**Week 4**				
N	40	40	36	36
Adjusted Mean Change from Baseline (SE) ‡	-9.06 (5.62)	-10.45 (5.10)	-7.31 (5.37)	-10.13 (5.68)
**Week 8**				
N	26	30	22	26
Adjusted Mean Change from Baseline (SE) ‡	-5.24 (5.96)	-1.69 (5.34)	-13.46 (5.82)	-11.57 (5.96)
**Week 12**				
N	21	26	17	20
Adjusted Mean Change from Baseline (SE) ‡	-2.18 (6.16)	-7.09 (5.47)	-10.47 (6.10)	-10.16 (6.23)
**Pain Change from Baseline**†				
**Week 4**				
N	40	40	36	36
Adjusted Mean Change from Baseline (SE) ‡	2.09 (6.13)	-2.07 (5.57)	1.52 (5.78)	2.82 (6.15)
**Week 8**				
N	26	30	22	26
Adjusted Mean Change from Baseline (SE) ‡	-1.61 (6.49)	-1.66 (5.83)	-1.08 (6.25)	2.49 (6.43)
**Week 12**				
N	21	26	17	20
Adjusted Mean Change from Baseline (SE) ‡	-5.95 (6.68)	-0.79 (5.94)	3.87 (6.55)	8.70 (6.72)

* The higher score indicates better “Global Health Status”.
† The lower score indicates lower level of “Pain”.
‡ The analysis used a mixed effects repeated measures model adjusted for treatment group, baseline score, ECOG PS (0+1 vs 2), previous treatment for metastatic PC (yes vs no), visit, country and visit by treatment interaction.
